# Association between daily glucose fluctuation and coronary plaque properties in patients receiving adequate lipid-lowering therapy assessed by continuous glucose monitoring and optical coherence tomography

**DOI:** 10.1186/s12933-015-0236-x

**Published:** 2015-06-11

**Authors:** Masaru Kuroda, Toshiro Shinke, Kazuhiko Sakaguchi, Hiromasa Otake, Tomofumi Takaya, Yushi Hirota, Tsuyoshi Osue, Hiroto Kinutani, Akihide Konishi, Hachidai Takahashi, Daisuke Terashita, Kenzo Uzu, Ken-ichi Hirata

**Affiliations:** Division of Cardiovascular Medicine, Department of Internal Medicine, Kobe, University Graduate School of Medicine, 7-5-1 Kusunoki-cho, Chuo-ku, Kobe, Hyogo 650-0017 Japan; Division of Diabetes and Metabolism, Department of Internal Medicine, Kobe, University Graduate School of Medicine, 7-5-1 Kusunoki-cho, Chuo-ku, Kobe, Hyogo 650-0017 Japan

**Keywords:** Glucose fluctuation, Continuous glucose monitoring, Mean amplitude of glycemic excursion, Optical coherence tomography, Thin-cap fibroatheroma

## Abstract

**Background:**

Glucose fluctuation has been recognized as a residual risk apart from dyslipidemia for the development of coronary artery disease (CAD). This study aimed to investigate the association between glucose fluctuation and coronary plaque morphology in CAD patients.

**Methods:**

This prospective study enrolled 72 consecutive CAD patients receiving adequate lipid-lowering therapy. They were divided into 3 tertiles according to the mean amplitude of glycemic excursions (MAGE), which represents glucose fluctuation, measured by continuous glucose monitoring (tertile 1; <49.1, tertile 2; 49.1 ~ 85.3, tertile 3; >85.3). Morphological feature of plaques were evaluated by optical coherence tomography. Lipid index (LI) (mean lipid arc × length), fibrous cap thickness (FCT), and the prevalence of thin-cap fibroatheroma (TCFA) were assessed in both culprit and non-culprit lesions.

**Results:**

In total, 166 lesions were evaluated. LI was stepwisely increased according to the tertile of MAGE (1958 ± 974 [tertile 1] vs. 2653 ± 1400 [tertile 2] vs. 4362 ± 1858 [tertile 3], p <0.001), whereas FCT was the thinnest in the tertile 3 (157.3 ± 73.0 μm vs. 104.0 ± 64.1 μm vs. 83.1 ± 34.7 μm, p <0.001, respectively). The tertile 3 had the highest prevalence of TCFA. Multiple linear regression analysis showed that MAGE had the strongest effect on LI and FCT (standardized coefficient β = 0.527 and −0.392, respectively, both P <0.001). Multiple logistic analysis identified MAGE as the only independent predictor of the presence of TCFA (odds ratio 1.034; P <0.001).

**Conclusions:**

Glucose fluctuation and hypoglycemia may impact the formation of lipid-rich plaques and thinning of fibrous cap in CAD patients with lipid-lowering therapy.

## Introduction

Dyslipidemia, especially a high low-density lipoprotein (LDL) cholesterol level, has been recognized as the most important promoter of atherosclerotic cardiovascular disease. A large number of clinical trials have reported beneficial effects of statins for primary and secondary prevention and improved all-cause mortality in association with lowering LDL cholesterol levels [1, 2]. However, the insufficiency of risk reduction with lipid-lowering therapy alone has accumulated attention on the unmet need for residual clinical risk management.

Diabetes mellitus (DM) is also a major risk factor for coronary artery disease (CAD) [3]. Several trials have demonstrated that hyperglycemia is relevant to the progression of atherosclerosis [4]. Recent investigations have revealed that compared with continuous hyperglycemia, large glucose fluctuation, such as postprandial hyperglycemia, might be one of the most deleterious factors in cardiovascular disease [5, 6]. Growing evidence further indicates that hypoglycemia has a negative impact on cardiovascular condition [7, 8]. With the recent emergence of continuous glucose monitoring (CGM) system, it has become possible to evaluate daily glucose fluctuation, including time in hyper- and hypoglycemia, in clinical practice. However, whether glucose fluctuation, including hypoglycemia, may have an impact on coronary plaque properties remains unclear.

Optical coherence tomography (OCT) is a high-resolution intravascular imaging modality that enables detailed assessment of coronary plaque character, such as lipid-rich, calcified, and fibrous, as well as fibrous cap thickness and vulnerable features . The aim of the present study was to investigate the relationship between glucose fluctuation and coronary plaque properties analyzed by CGM and OCT, respectively.

## Methods

### Patient population

**Fig. 1:**
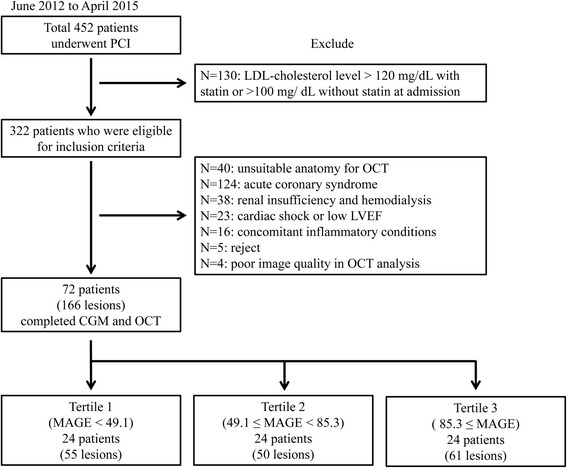
Study population. Seventy-two were enrolled in this study. CGM: continuous glucose monitoring; DM: diabetes mellitus; LDL-cholesterol: low-density lipoprotein-cholesterol; LVEF: left ventricular ejection fraction; OCT: optical coherence tomography; PCI: percutaneous coronary intervention

Seventy-two consecutive patients who had been referred for percutaneous coronary intervention (PCI) for coronary artery disease (CAD) during the period from June 2012 to April 2015 and who fulfilled the following inclusion criteria were enrolled in this prospective registry (Fig. 1): 20–80 years of age under adequate treatment of dyslipidemia; LDL cholesterol levels <120 mg/dL under statin administration or <100 mg/dL under other treatment for dyslipidemia including lifestyle management. The study exclusion criteria were 1) PCI for acute coronary syndrome; 2) unsuitable anatomy for OCT; 3) presented with cardiogenic shock or left ventricular ejection fraction <35 %; 4) concomitant inflammatory condition (such as active infection, inflammatory arthritis, or connective tissue disease) or malignancy; and 5) renal insufficiency with baseline creatinine level ≥2.0 mg/dL, including dependence on hemodialysis. We divided the patients into three groups according to the tertile of mean amplitude of glycemic excursions (MAGE, tertile 1; <49.1, tertile 2; 49.1 ~ 85.3, tertile 3; >85.3). MAGE, which was first proposed by Service et al., [9] represents fluctuations in blood glucose levels over a 24-h period and was calculated from the daily variations in blood glucose level, measured continuously by CGM over a period of 2 days.

This study was approved by the ethics committee of Kobe University and was carried out according to the guidelines of the Declaration of Helsinki. All enrolled study patients provided their written informed consent for enrolment into the study.

### Study protocol

**Fig. 2:**
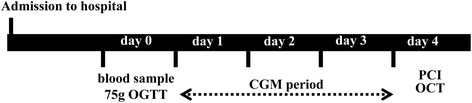
Study protocol. On admission, a blood sample analysis and 75-g oral glucose tolerance test (OGTT) were performed under fasting conditions. Subcutaneous interstitial glucose levels were monitored over a period of 3 consecutive days using continuous glucose monitoring (CGM). After the CGM examination, 3 vessels were subjected to optical coherence tomography (OCT) examination

On admission, a blood sample analysis was performed under fasting conditions to evaluate levels of creatinine, glycated hemoglobin (HbA1c), total cholesterol, LDL cholesterol, high-density lipoprotein cholesterol, triglycerides, and C-reactive protein. In addition, all patients underwent a 75-g oral glucose tolerance test (OGTT), and levels of plasma glucose and immunoreactive insulin were evaluated just before and 120 min after the oral glucose load. Subcutaneous interstitial glucose levels were monitored over a period of 3 consecutive days using the iPro™2 CGM system (Medtronic, Northridge, CA) (Fig. 2). After the CGM examination, three vessels were subjected to OCT examination, including both culprit lesions for PCI and non-culprit lesions. Culprit lesions were identified by analyzing pre-crisis and inter-crisis electrocardiograms, left ventricular wall motion abnormalities, and angiographic lesion appearance. Non-culprit lesions were defined as angiographically intermediate lesions (diameter of stenosis 30–70 %) with an identified plaque narrowing lumen area <4 mm^2^ by OCT.

### Continuous glucose monitoring system

CGM was performed for 3 consecutive days before PCI, and the daily glucose profile was analyzed using data obtained on days 2 and 3 to avoid any bias due to insertion or removal of the sensor. In all patients, CGM analysis software (CareLink iPro, Medtronic, Northridge, CA) calculated the median of the variables measured on days 2 and 3: 24-h mean glucose levels, time in hyperglycemia/hypoglycemia, and MAGE. Time in hyperglycemia and hypoglycemia was defined as the time when blood glucose levels were above 140 mg/dL and under 70 mg/dL, respectively. All patients received optimal meals (25–28 kcal/kg of ideal body weight; 60 % carbohydrate, 15–20 % protein, and 20–25 % fat) during CGM.

### OCT imaging

Images were acquired using a commercially available, frequency-domain OCT imaging system (ILUMIEN; St. Jude Medical Inc., St Paul, MN, USA). In this system, a 2.7-Fr OCT imaging catheter is advanced distal to the lesion, and automated pullback is initiated in concordance with blood clearance by the injection of contrast media. All images were de-identified and digitally stored.

### OCT analysis

**Fig. 3:**
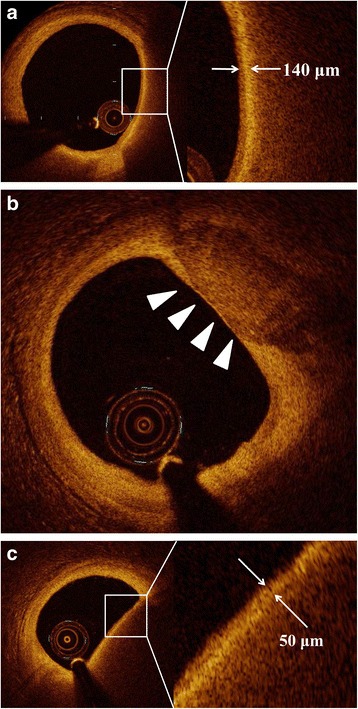
Representative optical coherence tomography images. **a** The representative case of lipid-rich plaque and the measurement of fibrous cap thickness. Lipid-rich plaque was defined as a diffusely bordered, signal-poor region (lipid pools) with overlying signal-rich bands, corresponding to fibrous caps. The fibrous cap thickness overlying a lipid-rich plaque was 140 μm at its thinnest part (*arrow*). **b** The representative case of calcification. Calcification was also recorded when an area consisted of a signal-poor or heterogeneous region with a sharply delineated border. **c** The representative case of thin-cap fibroatheroma (TCFA) The fibrous cap thickness of this lesion was 50 μm (*white arrows*), and this plaque was diagnosed as TCFA

All OCT images were analyzed by 2 independent investigators (MK, HO), who were blinded to the angiographic and clinical findings, using Off-line Review Workstation. When discordance in terms of qualitative plaque morphology occurred between observers, a consensus reading was obtained with third investigator (TS). Using OCT examination, a lipid-rich plaque was defined as a diffusely bordered, signal-poor region (lipid pools) with overlying signal-rich bands, corresponding to fibrous caps (Fig. 3a) [10]. The lipid arc was measured at every 1-mm interval throughout the length of each lesion, and the values were averaged. Lipid length was also measured on longitudinal view. Lipid index (LI) was defined as the mean lipid arc multiplied by lipid length [11]. Fibrous cap was defined as a signal-rich homogenous layer overlying the lipid-rich plaque [12]. The thinnest part of the fibrous cap was measured 3 times, and its average was defined as fibrous cap thickness (FCT) [13]. Calcification was also recorded when an area consisted of a signal-poor or heterogeneous region with a sharply delineated border (Fig. 3b) [14]. Calcification arc was measured at every 1-mm interval throughout the length of each lesion, and the values were averaged. Calcification length was also measured on longitudinal view. As with LI, calcification index (CI) was defined as the mean calcification arc multiplied by calcification length. Intraclass correlation coefficients for intra- and interobserver reliabilities were 0.982 and 0.958, respectively, for the lipid arc, and 0.992 and 0.963, respectively, for the calcification arc.

The presence of TCFA, macrophage infiltration, microchannels, and thrombus was also assessed. Thin-cap fibroatheroma (TCFA) was defined as a thin fibrous cap (<65 μm) overlying a lipid-rich plaque (>90°) (Fig. 3c) [15]. Microchannels were defined as signal-poor voids that were sharply delineated in multiple contiguous frames [14, 16]. Intracoronary thrombus was defined as a mass (diameter ≥ 250 μm) attached to the luminal surface or floating within the lumen, including red (red blood cell-rich) thrombus, which showed high backscattering with high attenuation (resembling blood), and white (platelet-rich) thrombus, which showed less backscattering, was homogeneous, and had low attenuation [14, 17]. Intraobserver and interobserver agreements for the detection of TCFA were within the acceptable range (intraobserver, kappa = 0.899; interobserver, kappa = 0.863).

### Statistical analysis

All data are presented as mean ± SD or proportions. Differences in continuous parameters among the 3 groups were calculated using a one-way analysis of variance for parametric data. The Tukey test was used as a post hoc analysis for continuous variables. Non-parametric parameters were compared using the Games–Howell test. Categorical variables were presented using frequency counts, and intergroup comparisons were analyzed by Fisher's exact test. Simple linear correlations were calculated using the concept of least squares and by determining the Pearson correlation coefficient, r. Multiple regression models were used to explore the influence of different variables on LI and FCT and to adjust for covariates. Multivariate logistic regression was performed to assess the impact of a set of factors on TCFA. Univariate analysis was performed first, and all variables that satisfied *P* < 0.2 were entered en bloc into the multivariate model, along with age, sex and medications as background variables. Intra-observer and interobserver reliabilities were estimated by the intraclass correlation coefficient for continuous measurement. To assess intraobserver and interobserver variabilities, categorical data were compared with the kappa test of concordance, and a Bland-Altman plot was used for continuous data. The sample size was estimated based on the previous report from Torimoto K, et al. [18]. They showed that glucose fluctuations in blood play a significant role in vascular endothelial dysfunction in 57 patients with type 2 diabetes in a sample size of 57 subjects provided power of 85 % at a significance level of 0.05 (r = 0.4) [18]. Referring to this report, we calculated the appropriate sample size. The association between glucose variability and plaque morphology in a sample size of 72 subjects provided power of 99.8 % at a significance level of 0.05 (r =0.50). Analyses were performed using commercially available software (IBM SPSS version 21, IBM Corp., Somers, New York).

## Results

### Baseline patient characteristics

Between June 2012 and April 2015, a total of 72 patients were enrolled. 85 % of the subjects presented with glucose intolerance, including newly and formerly diagnosed DM and impaired glucose tolerance. Baseline patient characteristics, medications on admission and variables measured by CGM are shown in Table 1. No significant differences among three group were observed in the baseline characteristics, except for the prevalence of DM and glycemic variables such as HbA1c, 1,5-AG, and glycoalbumin levels. According to the 75-g OGTT data, 2-h PG levels was significantly the highest in the tertile 3 among the three group. Medications on admission did not differ among the three groups, except for diabetes drugs usage. All variables obtained by CGM had significantly greater values in the tertile 3 than the other groups except for minimum blood glucose.

**Table 1:** Baseline patient characteristics

	Overall (*n* = 72)	Tertile 1 (MAGE <49.1) (*n* = 24)	Tertile 2 (49.1 ≤ MAGE <85.3) (*n* = 24)	Tertile 3 (85.3 ≤ MAGE) (*n* = 24)	P value
Age (years)	69.9 ± 11.3	69.2 ± 12.0	71.1 ± 9.8	69.5 ± 12.3	0.82
BMI (kg/m^2^)	24.1 ± 3.0	23.7 ± 2.6	24.5 ± 2.4	24.0 ± 3.9	0.64
Male	58 (80.6)	19 (79.2)	19 (79.2)	20 (83.3)	0.91
DM	36 (50.0)	6 (25.0)	11 (45.8)	19 (79.2)	0.001
Hypertension	53 (73.6)	19 (79.2)	16 (66.7)	18 (75.0)	0.61
Dyslipidemia	60 (83.3)	18 (75.0)	22 (91.7)	20 (83.3)	0.30
Smoking	49 (68.1)	15 (62.5)	16 (66.7)	18 (75.0)	0.52
Current	19 (26.4)	6 (25.0)	12 (50.0)	9 (37.5)	
Former (quit >3 months)	30 (41.7)	9 (37.5)	4 (16.7)	9 (37.5)	
Prior myocardial infarction	14 (19.4)	4 (16.7)	5 (20.8)	5 (20.8)	0.92
Prior PCI	33 (45.8)	11 (45.8)	12 (50.0)	14 (58.3)	0.85
Systolic blood pressure (mmHg)	121.5 ± 11.4	124.1 ± 11.2	121.4 ± 10.5	119.1 ± 12.4	0.32
Diastolic blood pressure (mmHg)	64.7 ± 9.0	64.2 ± 8.7	66.3 ± 9.3	63.5 ± 9.1	0.55
Left ventricular ejection fraction (%)	59.8 ± 9.6	57.5 ± 12.8	60.8 ± 8.7	61.1 ± 5.9	0.37
Duration of DM (years)	3.7 ± 7.7	0	3.5 ± 6.7	7.4 ± 10.5	0.003
HbA1c (NGSP) (%)	6.3 ± 1.0	5.8 ± 0.4	6.1 ± 0.6	6.9 ± 1.2	<0.001
1,5-AG (μg/mL)	15.8 ± 8.4	20.6 ± 10.2	15.5 ± 6.0	10.8 ± 5.3	<0.001
Glycoalbumin (%)	16.2 ± 3.5	14.6 ± 1.7	15.1 ± 2.7	19.2 ± 3.7	<0.001
75-g OGTT					
Fasting PG (mg/dL)	101 ± 23	89 ± 10	106 ± 29	107 ± 22	0.009
2-h PG (mg/dL)	206 ± 78	159 ± 45	209 ± 65	255 ± 90	<0.001
Fasting IRI (μU/mL)	6.9 ± 5.9	6.9 ± 5.3	9.0 ± 7.7	4.6 ± 3.0	0.044
2-h IRI (μU/mL)	83.6 ± 65.7	94.9 ± 59.9	92.0 ± 71.1	59.8 ± 62.6	0.156
HOMA R	1.8 ± 2.3	1.5 ± 1.1	2.7 ± 3.5	1.2 ± 0.7	0.056
HOMA β	82.5 ± 72.6	109.5 ± 95.0	83.3 ± 48.8	50.9 ± 53.8	0.024
Total cholesterol (mg/dL)	155.3 ± 27.9	161.8 ± 30.3	149.0 ± 21.3	154.9 ± 30.8	0.29
LDL cholesterol (mg/dL)	88.7 ± 19.1	88.5 ± 17.5	87.2 ± 17.4	90.4 ± 22.6	0.84
HDL cholesterol (mg/dL)	45.9 ± 11.3	46.1 ± 11.8	44.2 ± 11.0	47.5 ± 11.4	0.61
Triglyceride (mg/dL)	124.8 ± 57.9	125.8 ± 68.8	128.7 ± 51.4	119.8 ± 54.5	0.87
CRP (mg/dL)	0.21 ± 0.33	0.14 ± 0.23	0.18 ± 0.24	0.30 ± 0.47	0.25
Creatinine (mg/dL)	0.93 ± 0.28	0.91 ± 0.21	0.88 ± 0.20	1.00 ± 0.38	0.30
Medication on admission					
Aspirin	57 (79.2)	19 (79.2)	20 (83.3)	18 (75.0)	0.78
Thienopyridine	32 (44.4)	10 (41.7)	10 (41.7)	12 (50.0)	0.80
Statin	54 (75.0)	17 (70.8)	20 (83.3)	17 (70.8)	0.51
EPA	3 (4.2)	2 (8.3)	1 (4.2)	0 (0.0)	0.35
Ezetimibe	5 (6.9)	0 (0.0)	5 (20.8)	0 (0.0)	0.005
Fibrate	2 (2.8)	0 (0.0)	0 (0.0)	2 (8.3)	0.13
ACE-I/ARB	40 (55.6)	12 (50.0)	13 (54.2)	15 (62.5)	0.75
Beta-blocker	26 (36.1)	7 (29.2)	9 (37.5)	10 (41.7)	0.64
Insulin	3 (4.2)	0 (0.0)	0 (0.0)	3 (12.5)	0.044
Metformin	6 (8.3)	0 (0.0)	1 (4.2)	5 (20.8)	0.022
SU	9 (12.5)	0 (0.0)	2 (8.3)	7 (29.2)	0.007
α-GI	5 (6.9)	0 (0.0)	2 (8.3)	3 (12.5)	0.22
DPP4-I	12 (16.7)	0 (0.0)	5 (20.8)	7 (29.2)	0.020
Variables measured by the continuous glucose monitoring system					
MAGE (mg/dL)	70 ± 31	38 ± 8	66 ± 10	106 ± 19	<0.001
Mean BG (mg/dL)	132 ± 29	116 ± 9	127 ± 25	153 ± 35	<0.001
Max BG (mg/dL)	212 ± 55	165 ± 22	210 ± 48	262 ± 41	<0.001
Min BG (mg/dL)	77 ± 28	78 ± 14	72 ± 28	79 ± 36	0.70
Time in hyperglycemia (hours)	19.0 ± 15.4	7.6 ± 6.3	18.30 ± 14.8	31.6 ± 13.3	<0.001
Time in hypoglycemia (hours)	1.4 ± 2.7	0.3 ± 0.6	1.4 ± 1.9	2.4 ± 4.0	0.023

Values are mean ± SD or no. (%) 1,5 AG: 1,5 anhydroglucitol; 75 g OGTT: 75-g oral glucose tolerance test; α-GI: α-glucosidase inhibitor; ACE-I: angiotensin converting enzyme-inhibitor; ARB: angiotensin II receptor blocker; BG: blood glucose; BMI: body mass index; CABG: coronary artery bypass graft; CRP: C-reactive protein; DM: diabetes mellitus; DPP4-I: dipeptidyl peptidase-4 inhibitor; EPA: eicosapentaenoic acid; HbA1c: glycated hemoglobin; HDL cholesterol: high-density lipoprotein cholesterol; HOMA B: homeostasis model assessment beta; HOMA R: homeostasis model assessment ratio; IRI: immunoreactive insulin; LDL cholesterol: low-density lipoprotein cholesterol; MAGE: mean amplitude of glycemic excursion; PCI: percutaneous coronary intervention; PG: plasma glucose , Time in hyperglycemia and hypoglycemia were defined as the time when blood glucose levels were above 140 mg/dL and under 70 mg/dL, respectively

### Plaque characteristics obtained by OCT examination

**Fig. 4:**
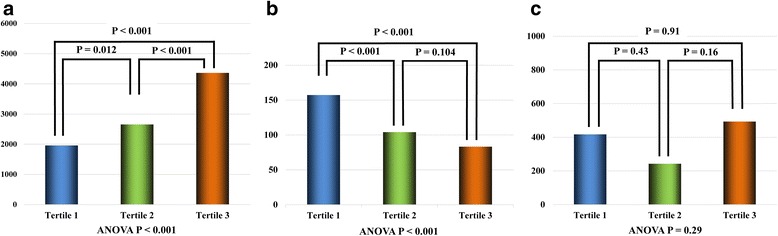
Comparison of OCT quantitative findings in plaque properties among 3 groups. **a** Lipid index (LI). **b** Fibrous cap thickness (FCT). **c** Calcification index (CI) LI was stepwisely increased according to the tertile of MAGE (1958 ± 974 [tertile 1] vs. 2653 ± 1400 [tertile 2] vs. 4362 ± 1858 [tertile 3], p <0.001, respectively) (**a**), whereas FCT was the thinnest in the tertile 3 (157.3 ± 73.0 μm vs. 104.0 ± 64.1 μm vs. 83.1 ± 34.7 μm, p <0.001, respectively) (**b**). CI did not differ among the three group (417 ± 968 vs. 242 ± 370 vs. 493 ± 842, p = 0.288, respectively) (**c**)

A total of 166 plaques including 64 culprit lesions and 102 non-culprit lesions were identified in 72 patients. The plaque characteristics are shown in Table 2. Tertile 3 had the highest prevalence of lipid-rich plaque, TCFA, and microchannel, whereas there was no difference in the frequency of calcification among three groups. Tertile 3 also had a significantly wider lipid arc, a longer lipid length, and a higher LI than other two groups, while CI did not differ among three groups (Fig. 4a and c). Also, FCT was significantly thinner in tertile 3 (Fig. 4b).

**Table 2:** Plaque characteristics

	Overall (n =166)	Tertile 1 (MAGE <49.1) (*n* =55)	Tertile 2 (49.1 ≤ MAGE <85.3) (*n* = 50)	Tertile 3 (85.3 ≤ MAGE) (*n* =61)	P value
Culprit lesion	64 (38.6)	20 (36.4)	21 (42.0)	23 (37.7)	0.83
Lipid-rich plaque	91 (54.8)	22 (40.0)	31 (62.0)	38 (62.3)	0.026
Lipid length (mm)	16.1 ± 6.7	13.2 ± 5.0	15.0 ± 6.2	19.5 ± 7.1	<0.001
Mean lipid arc	183.1 ± 51.9	147.1 ± 37.2	173.5 ± 43.7	223.4 ± 41.0	<0.001
Calcification	84 (50.6)	25 (45.4)	29 (58.0)	30 (49.2)	0.42
Calcification length (mm)	5.3 ± 3.8	5.6 ± 3.7	4.3 ± 2.7	6.1 ± 4.6	0.21
Mean calcification arc	119.6 ± 71.3	125.8 ± 73.0	90.5 ± 42.6	141.7 ± 83.2	0.019
TCFA	37 (22.3)	4 (7.3)	9 (18.0)	24 (39.3)	<0.001
Microchannel	99 (59.6)	23 (41.8)	29 (58.0)	47 (77.0)	0.001
Thrombus	13 (7.8)	1 (1.8)	5 (10.0)	7 (11.5)	0.12
Plaque location					0.79
RCA					
Proximal	28 (16.9)	8 (14.5)	9 (18.0)	11 (18.0)	
Mid	12 (7.2)	5 (9.1)	3 (6.0)	4 (6.6)	
Distal	8 (4.8)	1 (1.8)	5 (10.0)	2 (3.3)	
LAD					
Proximal	34 (20.5)	11 (20.0)	9 (18.0)	14 (22.9)	
Mid	27 (16.2)	8 (14.5)	10 (20.0)	9 (14.7)	
Distal	10 (6.0)	4 (7.3)	4 (8.0)	2 (3.3)	
LCx					
Proximal	13 (7.8)	7 (12.7)	1 (2.0)	5 (8.2)	
Mid	26 (15.7)	8 (14.5)	7 (14.0)	11 (18.0)	
Distal	8 (4.8)	3 (5.5)	2 (4.0)	3 (4.9)	

Values are mean ± SD or no. (%) LAD: left anterior descending artery; LCx: left circumflex artery; RCA: right coronary artery; TCFA: thin-cap fibroatheroma

### Association between OCT measurements and markers of diabetic control and nonglycemic metabolic variables

Lipid and calcification variables were tested for simple linear correlations against markers of glucose control and other laboratory variables (Table 3). MAGE had the strongest correlation with LI (r = 0.541; *P* < 0.001). Mean blood glucose, the time in hyperglycemia, HbA1c, and duration of DM were also observed to significantly correlate with LI. FCT was negatively correlated with MAGE and the time in hyper- and hypoglycemia, although there was no correlation between FCT and DM duration. LI and FCT did not correlate with other clinical or laboratory variables. Calcification length and CI did not correlate with any diabetic or nonglycemic variables, except a significant positive correlation between CI and DM duration.

**Table 3:** Pearson correlation coefficients, association between OCT measurements and markers of diabetic control and nonglycemic metabolic variables

	MAGE	Mean BG	Time in hyperglycemia	Time in hypoglycemia	HbA1c	DM duration	LDL cholesterol	HDL cholesterol	TG	CRP
Lipid length	0.369**	0.292**	0.349**	0.071	0.173*	0.193*	−0.065	−0.044	0.019	−0.061
Lipid mean arc	0.615**	0.324**	0.397**	0.232**	0.330**	0.268**	0.173	−0.017	−0.018	0.286**
Lipid index	0.541**	0.340**	0.405**	0.149	0.268*	0.257**	0.043	−0.053	0.037	0.068
Fibrous cap thickness	−0.430**	−0.105	−0.192**	−0.316**	−0.097	−0.046	−0.039	0.113	0.041	−0.149
Calcification length	0.099	0.065	0.149	0.132	−0.105	0.102	0.171	0.094	−0.053	0.026
Calcification mean arc	0.154	0.236*	0.253*	0.040	−0.022	0.184	0.159	0.081	−0.029	−0.171
Calcification index	0.101	0.099	0.166*	0.085	−0.047	0.227**	−0.005	0.143	−0.034	−0.108

*P < 0.05; **P < 0.001. BG: blood glucose; CRP, C-reactive protein; DM: diabetes mellitus; HbA1c: glycated hemoglobin; HDL cholesterol: high-density lipoprotein cholesterol; LDL cholesterol: low-density lipoprotein cholesterol; MAGE: mean amplitude of glycemic excursion

Multiple linear regression analysis was performed to assess the independent effects of diabetic parameters on LI and FCT (Table 4). Multivariate analysis demonstrated that a larger MAGE was independently associated with a higher LI and thinner FCT (standardized coefficient β = 0.527 and −0.392, respectively; both *P* < 0.001). Also, in multivariate analysis, longer duration of hypoglycemia significantly correlated with thinner FCT.

**Table 4:** Linear multivariate analyses using (A) lipid index and (B) fibrous cap thickness as the dependent variable, (1) In all patients (*n* = 166), (2) in DM patients (*n* = 83), and (3) in non-DM patient (*n* = 83)

(1) in all patients					
(A)					
Variables	Non-standardized coefficients		Standardized coefficients β	t	P value
	B	SE			
MAGE	31.9	4.239	0.527	7.541	<0.001
Mean BG	10.4	5.075	0.143	2.051	0.042
(B)					
MAGE	−0.868	0.171	−0.392	−5.076	<0.001
Time in hypoglycemia	−3.789	1.828	−0.160	−0.160	0.040
(2) in DM groups					
(A)					
MAGE	35.0	6.554	0.515	5.346	<0.001
(B)					
MAGE	−0.906	0.212	−0.433	−4.265	<0.001
(3) in non-DM patients					
(A)					
MAGE	31.9	5.633	0.532	5.661	<0.001
(B)					
MAGE	−1.357	0.280	−0.474	−4.847	<0.001

BG: blood glucose; MAGE: mean amplitude of glycemic excursion

### Association between TCFA and clinical and laboratory variables

Multivariate analysis was performed to identify independent risk factors for TCFA (Table 5). Univariate analysis was first conducted to identify potential factors, such as medications, glucose control and other laboratory variables, for TCFA. All variables with *P* < 0.2 on univariate analysis were tested by multivariate analysis. In all plaques, MAGE was the only independent predictor of TCFA (odds ratio 1.034, 95 % confidence interval 1.019–1.049, *P* < 0.001).

**Table 5:** Univariate and multivariate logistic regression analyses for determinants of TCFA

Variables	Univariate			Multivariate		
	OR	95 % CI	P value	OR	95 % CI	P value
MAGE	1.028	1.014–1.041	<0.001	1.034	1.019–1.049	<0.001
HbA1c	1.470	1.034–2.091	0.032			
1,5-AG	0.940	0.888-0.996	0.035			
Time in hypoglycemia	1.105	0.983-1.242	0.093			

1,5 AG: 1,5 anhydroglucitol; CI: confidence interval; HbA1c: glycated hemoglobin; MAGE: mean amplitude of glycemic excursion; OR: odds ratio

### DM vs. non-DM patients

**Fig. 5:**
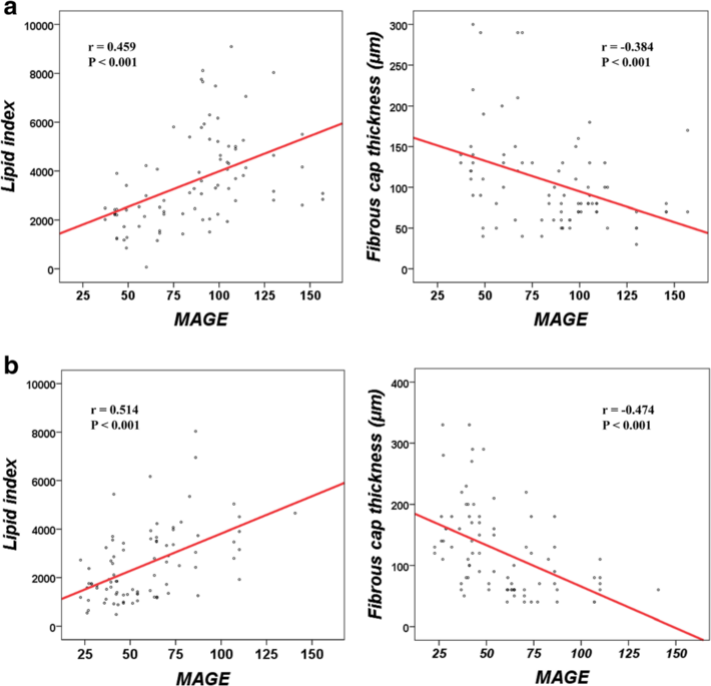
The correlation between mean amplitude of glycemic excursion (MAGE) and lipid index (LI) and fibrous cap thickness (FCT). This figure shows the correlation between MAGE and LI and FCT in **a** DM and **b** non-DM groups

We divided the patients into two groups according to whether or not they met the DM criteria (DM group and non-DM group) and the correlation with MAGE and OCT findings assessed in two groups. We detected 83 plaques in 36 DM patients (2.3 plaques/patient) and 83 plaques in 36 non-DM patients (2.3 plaques/patient). In both the DM group and the non-DM group, MAGE significantly correlated with LI and FCT (DM group; r = 0.459 and −0.384, both *P* < 0.001, non-DM group; r = 0.514 and −0.474; both *P* < 0.001, respectively) (Fig. 5). In both groups, multivariate analysis demonstrated that larger MAGE was significantly associated with higher LI and thinner FCT (Table 4).

## Discussion

The current study represents an in vivo report to assess the impact of glucose fluctuations on coronary plaque properties using CGM and OCT in patients with CAD and under statin administration or other dyslipidemia management. The novel findings of this study can be summarized as follows: 1) in CAD patients under adequate treatment for dyslipidemia, glucose fluctuation might be an important contributing factor to the formation of lipid-rich plaque; 2) a longer time in hypoglycemia was significantly correlated with a thinner FCT; and 3) coronary plaque calcification was associated with the duration of DM rather than daily glucose fluctuation.

### Association between glucose fluctuation and development of coronary artery disease

The present study showed that glucose fluctuation had a stronger correlation with LI and FCT than any other variables, based on OCT examination. Several in vitro investigations have reported that glucose variability might be involved in the development of oxidative stress and vascular injury, compared with exposure to constant high glucose [18, 19]. Schisano et al. reported that in vitro, prolonged exposure to oscillating glucose generated a more detrimental condition in terms of oxidative stress and DNA damage for endothelial cells than constant high glucose, caused by hyperactivation of p53. Other in vivo investigations also suggested that glucose fluctuation may be involved in the development of vascular injury, compared with constant high blood glucose [5, 20, 21]. Mita et al. demonstrated that oscillations in blood glucose concentrations accelerated macrophage adhesion to the endothelial surface and the formation of arteriosclerotic lesions independent of lipid profile in apolipoprotein E-deficient mice [20]. Teraguchi I, et al. reported that glycemic fluctuation assessed by MAGE is significantly associated with coronary plaque rupture in patients with primary acute myocardial infarction [21]. The current study also reported that compared with a marker of averaged blood glucose level such as HbA1c, MAGE was a more contributing factor of plaque vulnerability. These investigations suggest that daily glucose fluctuation could have a more ominous impact on the function of endothelial cells to promote atherosclerosis, leading to the advancement of plaque vulnerability. Meanwhile, the present study found no relationship between glucose fluctuation and coronary calcification. Although DM patients have been recognized to have a higher incidence of coronary calcification than non-DM patients, few investigations have reported that glucose fluctuation also could be associated with coronary calcification. Therefore, future studies are needed to further elucidate this point.

Several previous studies reported that oxidative stress is not related to glucose fluctuation in type 1 diabetes [22, 23]. On the other hand, some studies investigated that glucose fluctuations exhibited a more specific triggering effect on oxidative stress than chronic sustained hyperglycemia in type 2 diabetes [5]. In the present study, patients with type 1 diabetes were not included. An explanation for the lack of correlation between glucose fluctuation and oxidative stress may be that, unlike those with type 2 diabetes, those with type 1 diabetes are not sensitive to glucose variability as a stimulator of oxidative stress, because of different underlying pathophysiological conditions, such as dyslipidemia, smoking, obesity and other risk factors [23].

### The impact of hypoglycemia on coronary plaque vulnerability

In the present study, the longer time in hypoglycemia was associated with a thinner FCT, suggesting that hypoglycemia may have an association with plaque vulnerability. The ACCORD trial revealed that severe hypoglycemia was strongly associated with increased risks of adverse clinical outcomes [7]. To date, hypoglycemia has been recognized to induce the activation of the sympathetic nervous system [8], followed by elevated blood pressure and vasoconstriction [24]. Gimenez et al. suggested that repeated hypoglycemic episodes are associated with a worsening of subclinical atherosclerosis profile represented not only by abnormalities in endothelial function but also by an increase in intima-media thickness [25]. Furthermore, a recent in vitro study reported that repetitive hypoglycemia enhanced monocyte adhesion to endothelial cells through enhanced adrenaline activity [26]. These observations suggest hypoglycemia might have some effects on the progression of vulnerable plaques. Moreover, although these data lead to the hypothesis that inhibition of glucose fluctuation is potentially an important intervention in the prevention of cardiovascular disease, a large-scale prospective study is warranted to evaluate whether additional glucose fluctuation control, including hypoglycemia, will decrease the progression of plaque vulnerability and late clinical events.

### Limitations

This study has several limitations. First, as a single-center study with a relatively small number of patients, a potential risk of patient selection bias exists. Second, we only assessed patients with stable angina pectoris. We excluded ACS patients from this study because those population was highly heterogeneous with multiple confounding factors, such as uncontrolled dyslipidemia, stress hyperglycemia, drastic changes in diet and medications after admission. Further study is warranted including ACS patients since those might be a better target to address the influence of MAGE on plaque characteristics because vulnerable plaques are more frequently found. Third, we did not validate the OCT findings with histological findings; therefore, the regions identified as TCFA were not confirmed to contain a large necrotic core overlapping with a thin cap. Fouth, we excluded patients whose LDL cholesterol level was >120 mg/dL under statin treatment or >100 mg/dL without statins and included only patients whose dyslipidemia was thought to be well controlled. To explore the direct effect of glucose fluctuation on coronary vulnerability, further study is warranted to include only patients with more strictly controlled dyslipidemia; i.e., LDL <70 mg/dL. Finally, in the present study, we could not find that the effects of anti-atherosclerotic drugs, such as statins, α-GI, DPP-4 inhibitors were not independently associated with the regression of plaque. Although we could not find the impact of anti-atherosclerotic drugs on the regression of lipid-rich plaque in this study, further study is warranted to evaluate whether additional glucose fluctuation control will decrease the progression of TCFA and late clinical events.

## Conclusions

In this OCT and CGM study focusing on the impact of glucose fluctuation on plaque properties, daily glucose fluctuation appeared to have an impact on the formation of lipid-rich plaque, including TCFA, rather than calcification. Moreover, longer time in hypoglycemia may have some associations on the formation of lipid-rich plaques, thus supporting the concept that hypoglycemia can be one of the mechanisms of plaque rupture. A large-scale, randomized study is warranted to validate whether early detection and control of glucose fluctuation, especially hypoglycemia, could be beneficial to improve the clinical outcome of patients with CAD receiving adequate lipid-lowering therapy.

## Abbreviations

CAD: Coronary artery disease
CGM: Continuous glucose monitoring
DM: Diabetes mellitus
FCT: Fibrous cap thickness
LI: Lipid index
MAGE: Mean amplitude of glycemic excursion
OCT: Optical coherence tomography
PCI: Percutaneous coronary intervention
TCFA: Thin-cap fibroatheroma
